# Incidence and risk factors of portomesenteric venous thrombosis after colorectal surgery for cancer in the elderly population

**DOI:** 10.1186/s12957-019-1739-9

**Published:** 2019-11-19

**Authors:** Michele Manigrasso, Marco Milone, Nunzio Velotti, Sara Vertaldi, Pietro Schettino, Mario Musella, Giovanni Aprea, Nicola Gennarelli, Francesco Maione, Giovanni Sarnelli, Pietro Venetucci, Giovanni Domenico De Palma, Francesco Milone

**Affiliations:** 1 Department of Clinical Medicine and Surgery, University “Federico II” of Naples, Naples, Italy; 2 Department of Gastroenterology, Endocrinology and Surgical Endoscopy, University of Naples “Federico II”, Via Pansini 5, 80131 Naples, Italy; 3 Department of Advanced Biomedical Sciences, University “Federico II” of Naples, Naples, Italy

**Keywords:** Portomesenteric, Venous thrombosis, Colorectal, Surgery

## Abstract

**Background:**

Although it is known that portomesenteric venous thrombosis (PMVT) is associated with total colectomy and proctocolectomy in young patients with inflammatory bowel disease, little is known about incidence and risk factors of PMVT among the elderly population undergoing colorectal surgery for cancer.

**Methods:**

Data of elderly patients (> 70 years) undergoing surgery for colorectal cancer were retrospectively registered. The occurrence of PMVT was correlated with the patients’ characteristics and operative variables. Data collected included age, sex, obesity, ASA score, tumor degree, type of surgical resection, surgical approach (laparoscopic or open), and duration of surgery (from skin incision to the application of dressings).

**Results:**

A total of 137 patients > 70 years who underwent surgery for colorectal cancer and developed an acute intraabdominal process with suggestive symptoms, needing a CT scan, were included. Three of these patients (2.1%) had portomesenteric venous thrombosis during the study period, which was proved with CT scan. There were no significant patients’ characteristics or operative variables between patients with or without the occurrence of PMVT after surgery. Of interest, only operative time was significantly higher in patients with PMVT after surgery (256 ± 40 vs 140 ± 41, *p* < 0.001).

**Conclusions:**

PMVT as a cause of abdominal pain after colorectal surgery for cancer in the elderly population is uncommon. An index of suspicion for PMVT in an elderly postoperative colorectal cancer patient with sudden onset of abdominal pain must be maintained.

## Background

Portomesenteric venous thrombosis (PMVT) is an uncommon, potentially life-threatening, condition. Data about PMVT in broader surgical population are scarce. Incidence and risk factors for PMVT development after major abdominal surgery, in particular colon and rectal procedures, have been recently studied [1, 2]. The incidence of postoperative PMVT has been reported to range from 2.8 to 9%. Although it is known that splanchnic venous thrombosis is associated with total colectomy and proctocolectomy in young patients with inflammatory bowel disease [3–5], little is known about incidence and risk factors of PMVT among the elderly population undergoing colorectal surgery for cancer. The incidence of postoperative PMVT after colorectal surgery has been reported to range from 2.8 to 9%. Factors reported to be associated with the development of PMVT include manipulation of mesenteric vessels, postoperative septic complications, and various underlying diseases.

The aim of this study is to evaluate the incidence of PMVT after colorectal surgery in the elderly as well as the presence of risk factors, presenting symptoms, and the treatment.

## Methods

Data of patients collected during a 12-year period (from September 2002 to July 2014) with diagnosis of colorectal cancer were retrospectively reviewed. The inclusion criteria were patients > 70 years who underwent surgical intervention and needed CT scan for the new onset of abdominal pain. CT scans were performed using a spiral CT scanner with intravenously administered contrast.

Our perioperative prophylaxis routinely included subcutaneous heparin administration and perioperative handling of antiplatelet drugs according to the current literature [6–9]. A complete coagulation profile was performed whenever a diagnosis of portomesenteric venous thrombosis was made. The coagulation profile included platelet count, thrombin time, antithrombin III, plasminogen, protein C, protein S, homocysteine level, factor V Leiden mutation, and prothrombin G20210A mutation,

Data collected included age, sex, obesity, ASA score, tumoral stage, type of surgical resection, surgical approach (laparoscopic or open), and duration of surgery (from skin incision to the application of dressings).

The occurrence of portomesenteric venous thrombosis was correlated with the patients’ characteristics and operative variables.

Statistical analysis was performed with SPSS 16.0. The Yates corrected *χ*^2^ test was used to evaluate differences in categorical variables, and the independent samples *t* test was used to analyze continuous variables. To adjust for covariates and to make predictions, logistic regression model was used. Statistical significance was accepted when the *p* value was less than 0.05.

## Results

A total of 137 patients > 70 years who underwent surgery for colorectal cancer with a postoperative onset of symptoms suggestive of an acute intraabdominal process, who therefore needed a CT scan were identified.

Three of these patients (2.1%) had portomesenteric venous thrombosis during the study period, which was proved with CT scan. In all three cases, splanchnic venous thrombosis could be identified by CT scan. In most cases, the main symptom was the new-onset of acute abdominal pain. Other signs and symptoms included fever in two patients, of which one presented an elevated white blood cell count, and nausea in one patient. The intercurrent time between surgery and the acute event was respectively 35, 62, and 80 days.

Patients’ characteristics and operative variables of entire study population and of the patients with PMVT are respectively shown in Tables 1 and 2.

**Table 1:** Patients characteristics and operative variables of the 137 patients with abdominal pain

Age (years)	75.5 ± 7.1
Male gender (pts)	70 (51%)
Obesity (pts)	27 (19.7%)
ASA score	
II	61 (44.5%)
III	53 (38.6%)
IV	23 (16.7%)
Tumor grade	
I	36 (26.2%)
II	58 (42.2%)
III	29 (21.1%)
IV	14 (10.2%)
Surgery (pts)	25 (18.2%)
Right colectomy	78 (56.9%)
Left colectomy	23 (16.7%)
Rectal resection	11 (8%)
Miles intervention	
Laparoscopy (pts)	34 (24.8%)
Operative time (min)	143.3 ± 44.8

**Table 2:** Patients with PMVT

	Age	Sex	Obesity	ASA	Tumor grade	Surgery	Approach	Operative time
Patient 1	75	Male	No	II	III	Right colectomy	Open	250
Patient 2	77	Female	NO	II	I	Miles	Open	220
Patient 3	82	Male	Yes	III	II	Rectal resection	Laparoscopic	300

Considering thrombosis risk factors, none of the three patients with PMVT had any alteration of coagulation profile.

All three patients recovered from the acute event, with no need for further surgical intervention. They have been discharged from the hospital with long-term systemic oral anticoagulation for a minimum of 6 months. In details, to all subjects were given intravenous fluids, prophylactic antibiotic therapy (3rd generation cephalosporin–ceftriaxone- 1 g e.v. twice daily) and low molecular weight heparin (LMWH) therapy (100 UI/Kg s.c. twice/day). A week later, 2 patients were given warfarin with a target PT INR value ≥ 2. The other patient underwent percutaneous transhepatic thrombolysis and mechanical thrombectomy prior to starting warfarin with a target PT INR value ≥ 2. In both cases, LMWH was stopped when PT INR reached values of INR ≥ 2 [10]. All the three cases of PMVT were successfully resolved.

After a median follow-up of 24 months, all three patients had discontinued taking their oral anticoagulants but no cases of recurrent thrombotic events have been reported. During the follow-up period, no death was reported.

There were no significant patients’ characteristics or operative variables between patients with or without the occurrence of PMVT after surgery. Of interest, only operative time was significantly higher in the group of patients with PMVT after surgery (256 ± 40 vs 140 ± 41, *p* < 0,001). After adjusting for patients’ characteristics and operative variables in a multivariate logistic regression, operative time showed a clear trend toward a higher PMVT occurrence (OR = 4.7 95% CI).

## Discussion

Life-threatening complications caused by thrombosis of portomesenteric venous system have been recognized and treated since the end of nineteenth century [11–13].

Portomesenteric venous thrombosis is known to be a rare condition that occurs in association with many other precipitating factors, capable of producing a wide range of clinical problems. Among the most common causes, there are hepatocellular carcinoma, cirrhosis, pyophlebitis from bowel disease, and spontaneous thrombosis from hypercoagulable state [14–17].

In this study, no patients had risk factors for thrombosis. In addition, there are no laboratory-chemical parameters specifically designed to confirm PMVT.

However, it is important to underline that the analysis of hematic parameters of thrombosis represents only a superficial examination, and it is not possible to rule out severe coagulation disorders with just this test.

At the moment, many medical centers perform CT scan as the primary imaging modality in patients with acute abdominal pain. CT scan is remarkably useful to identify bowel perforation or obstruction, as well as venous thrombosis or arterial ischemia [18–21].

PMVT development after colorectal surgery has been recently studied. This surgical population can include many of the proposed PMVT risk factors.

Several conditions can be cause of PMVT after colorectal surgery: the type of procedure, intraoperative manipulation of mesenteric vessels, intraabdominal pressure created by a pneumoperitoneum, intraabdominal septic complications, inherited or acquired prothrombotic conditions [2].

In fact, patients suffering from IBDs and cancer have a higher risk for venous thrombotic events, and this risk is further increased by septic complications and surgery [15, 17].

As far as we know, this is the first multicentric institution series evaluating the incidence and risk factors of symptomatic PMVT in elderly patients, undergoing elective colorectal surgery for cancer, with both open and laparoscopic technique.

It is well known that less invasive approach reached in the last decades increased popularity in each surgical field and it is considered the best surgical approach to colorectal and gastric malignancy [22–32]: shorter recovery and hospitalization, lower postoperative pain, and a better cosmetic results comparing with the open surgery are the main advantages of this technique, even in the elderly population [33, 34].

On the other side, data about PMWT after laparoscopic procedures are scarce.

Up to now, only Allaix et al. [2] retrospectively analyzed 1069 consecutive laparoscopic colorectal resections for inflammatory bowel disease and colorectal cancer. 3.5% patients experienced symptomatic postoperative PMVT. Robinson et al. [1] retrospectively reviewed 1224 patients who underwent colorectal surgery for carcinoma, IBD, or diverticulitis. Three percent of them were diagnosed with PMVT. Remzi et al. [35] retrospectively reviewed 702 patients undergoing total proctocolectomy and ileal pouch anal anastomosis for ulcerative colitis by an open approach. Symptomatic PMVT was observed in 6% of cases. Also Fichera et al. [3] published similar results in a retrospective analysis of 83 consecutive patients undergoing open total colectomy for IBD. They found a symptomatic PMVT in about 5% of the patients studied. Finally, Antiel RM et al. [4] reviewed 366 pediatric patients who underwent colectomy for ulcerative colitis. Four percent were diagnosed with PMVT.

Thus, it is known that splanchnic venous thrombosis is associated with total colectomy and proctocolectomy in young patients with inflammatory bowel disease.

However, little is known about the incidence of PMVT after colorectal surgery in the elderly population. Mean age among current literature varies from 17.9 to 62 years [2, 4].

Speaking of incidence of PMVT, our observation confirms the findings of the other studies published in the literature; moreover, it extended these results to the elderly population.

We found symptomatic PMVT in 2.1% of the elderly (> 70 years) population (mean age of 75.5 ± 7.1 years) after surgery. No death occurred during the 24-month follow-up period. Thus PMVT is not an uncommon cause of abdominal pain after colorectal surgery for cancer in the elderly population.

Risk factors for PMVT identified with univariate and multivariate analysis include only operative time. Of interest, laparoscopic colorectal resections were not associated with a higher risk of portomesenteric venous thrombosis occurrence.

Some limitations of this study have to be addressed. First of all, the study design, which is retrospective; another limitation is the small sample size, which could not be a guarantee of correlation between PMVT and surgical procedure; finally, the long period between discharge and PMVT that makes difficult the correlation between the intervention and the adverse event, although this is probably suggested by the lack of other risk factors.

## Conclusions

PMVT is a complication of colorectal surgery, especially in the elderly population. Of our 137 patients, only three of them were symptomatic and present no specific symptoms.

CT scan was the best tool to investigate the presence of PMVT and confirmed in all cases the splanchnic vein thrombosis.

Although being this study a retrospective analysis, further studies are needed to confirm these results, an index of suspicion for PMVT in an elderly postoperative colorectal cancer patients with sudden onset of abdominal pain and fever must be maintained, especially in case a septic cause cannot be identified.

## Data Availability

All data are available and stored by the authors.

## Abbreviations

CT: Computed tomography
IBD: Intestinal bowel disease
INR: International normalized ratio
LMWH: Low molecular weight heparin
PMVT: Portomesenteric vein thrombosis
SMV: Spleno mesenteric vein
